# Potassium Alleviates Post-anthesis Photosynthetic Reductions in Winter Wheat Caused by Waterlogging at the Stem Elongation Stage

**DOI:** 10.3389/fpls.2020.607475

**Published:** 2021-01-12

**Authors:** Jingwen Gao, Yao Su, Man Yu, Yiqian Huang, Feng Wang, Alin Shen

**Affiliations:** Environmental Resources and Soil Fertilizer Institute, Zhejiang Academy of Agricultural Sciences, Hangzhou, China

**Keywords:** waterlogging, potassium, photosynthesis, senescence, winter wheat

## Abstract

Waterlogging occurs frequently at the stem elongation stage of wheat in southern China, decreasing post-anthesis photosynthetic rates and constraining grain filling. This phenomenon, and the mitigating effect of nutrient application, should be investigated as it could lead to improved agronomic guidelines. We exposed pot-cultured wheat plants at the stem elongation stage to waterlogging treatment in combination with two rates of potassium (K) application. Waterlogging treatment resulted in grain yield losses, which we attributed to a reduction in the 1,000-grain weight caused by an early decline in the net photosynthetic rate (Pn) post-anthesis. These decreases were offset by increasing K application. Stomatal conductance (*G*_s_) and the intercellular CO_2_ concentration (*C*_i_) decreased in the period 7–21 days after anthesis (DAA), and these reductions were exacerbated by waterlogging. However, in the period 21–28 DAA, *G*_s_ and *C*_i_ increased, while Pn decreased continuously, suggesting that non-stomatal factors constrained photosynthesis. On DAA 21, Pn was reduced by waterlogging, but photochemical efficiency (Φ*_PSII_*) remained unchanged, indicating a reduction in the dissipation of energy captured by photosystem II (PSII) through the CO_2_ assimilation pathway. This reduction in energy dissipation increased the risk of photodamage, as shown by early reductions in Φ*_PSII_* in waterlogged plants on DAA 28. However, increased K application promoted root growth and nutrient status under waterlogging, thereby improving photosynthesis post-anthesis. In conclusion, the decrease in Pn caused by waterlogging was attributable to stomatal closure during early senescence; during later senescence, a reduction in CO_2_ assimilation accounted for the reduced Pn and elevated the risk of photodamage. However, K application mitigated waterlogging-accelerated photosynthetic reductions and reduced yield losses.

## Introduction

About 15–20% of global wheat production is affected by waterlogging every year, and this proportion is rising due to frequent extreme weather events during the ongoing global warming process (Trnka et al., 2014; Herzog et al., 2016; Manik et al., 2019). Waterlogging occurs when pores in the soil fill with water due to heavy rainfall and poor soil drainage. Waterlogging causes hypoxia in the rhizosphere, which inhibits root energy production through aerobic respiration. Wheat is a dry land crop that is extremely sensitive to waterlogging stress. Grain yields are reduced by 20–50% when wheat is subjected to this form of stress (Singh and Setter, 2017; Nguyen et al., 2018). The rice–wheat rotation system commonly used in southern China causes soil compaction, which exacerbates waterlogging stress. Waterlogging is especially frequent in the region during springtime when rains are heavy. The key growth periods of wheat from the stem elongation to booting stages occur in this season (Chen et al., 2018). Waterlogging during these developmental stages reduces wheat yield to a greater extent than excessive rainfall in others (Celedonio et al., 2014). Waterlogging between the stem elongation and booting stages is a crucial factor constraining wheat production.

Waterlogging at the stem elongation stage accelerates the decrease in net photosynthetic rate (Pn) post-anthesis, slows grain filling, and reduces the 1,000-kernel weight (Araki et al., 2012; Romina et al., 2018; Wollmer et al., 2018). There have been few investigations of the key factors underlying the early decline in Pn post-anthesis during periods of waterlogging. This decline in Pn is an indicator of leaf senescence, which is caused by a combination of mechanisms, including the degradation of photosynthetic nitrogenous compounds, stomatal closure, a reduction in photochemical efficiency, and the generation of free radicals (Masclaux et al., 2000). Camp et al. (1982) found that during leaf senescence, the reduction of Pn is early to the degradation of photosynthetic nitrogenous compounds. Besides, stomata are sensitive to environmental and physiological changes and play an important role in regulating leaf senescence (Satler, 1979; Thimann and Satler, 1979; Wang et al., 2019). Stomatal closure may be responsible for the reduction in Pn seen during the early stage of leaf senescence, but this requires confirmation by experimental analysis. The degradation of nitrogenous compounds contributes further reductions in photochemical efficiency. Nitrogen (N) deficiency also accelerates N recycling and mobilization during leaf senescence (Diaz et al., 2008). Waterlogging restricts root energy production and extension growth, which, in turn, limits the absorption of water and nutrients (Herzog et al., 2016). Therefore, waterlogging may inhibit photosynthesis by restricting stomatal function and decreasing photochemical efficiency. The dynamic responses of photosynthetic factors post-anthesis during waterlogging events warrant further study.

Nutrient management is an efficient way of mitigating the impact of abiotic stress on plant growth and yield formation development (Noreen et al., 2018). Potassium (K) is an essential macronutrient that is not used in organic matter synthesis like N and phosphorus (P). K is involved in a variety of physiological mechanisms. It can regulate guard cell movements to promote stomatal opening (Tränkner et al., 2018). K also participates in energy production (Cui et al., 2019) and long-distance transport of carbohydrates to the roots (Dreyer et al., 2017; Tränkner et al., 2018), which may regulate root growth and nutrient uptake during waterlogging. Hence, the application of K may mitigate the waterlogging-accelerated decrease in Pn post-wheat anthesis through stomatal mechanism and improvements in leaf nutrients. The effects of K should be further investigated to provide famers with improved procedures for dealing with waterlogging in their fields.

We performed a pot experiment to study (i) the effects of waterlogging at the stem elongation stage on photosynthetic responses post-anthesis and (ii) the effects of K application. We postulated that waterlogging at the stem elongation stage would lead to stomatal closure, initiating a decline in the photosynthetic rate post-anthesis, and nutrient deficiency would further reduce Pn. We also postulated that the application of K would reduce the inhibitory effects of waterlogging on photosynthesis post-anthesis and yield development.

## Materials and Methods

### Plant Materials and Growth Environment

Our plant material was the Yangmai20 wheat variety, which is used widely in southern wheat growing areas of China. We produced seeds in our laboratory from the material acquired from the Jiangsu Academy of Agricultural Sciences. The pot experiment was conducted in a greenhouse at the Experimental Research Station of the Zhejiang Academy of Agricultural Sciences, Hangzhou, China. The experiment was performed during the growing season of 2019–2020. Wheat seeds were surface-sterilized with a 0.5% hypochlorite solution before sowing. Seeds were sown in plastic pots (22 cm in height, 25 cm in diameter), each with a drain hole in the bottom. Air-dried and sieved (0.5 mm mesh) clay loam and river sand were mixed in a 2:1 ratio; each pot was filled with 8 kg of this mixture. The soil pH, organic matter content, bulk density, field capacity (FC) by volume, total N, available P, and available K were 6.7, 4.2%, 1.1 g cm^–3^, 41.2%, 1.8 g kg^–1^, 39.2 mg kg^–1^, and 115.2 mg kg^–1^, respectively. We planted 15 seeds in each pot, and the seedlings were thinned to seven per pot at the two-leaf stage (Zadoks Demical code 13). The soils were irrigated with tap water to 70–80% FC until the start of waterlogging treatment.

### Experimental Design

Two water treatments (well-watered and waterlogged) and two K application rates (0.375 and 0.75 g K pot^–1^, designated as the conventional K rate and the increased K rate, respectively) were applied to the plants. Four treatment combinations were established: well-watered + 0.375 g K pot^–1^ (control, C), well-watered + 0.75 g K pot^–1^ (C + K), waterlogged + 0.375 g K pot^–1^ (W), and waterlogged + 0.75 g K pot^–1^ (W + K). The waterlogging treatment was applied at the stem elongation stage (Zadoks Demical code 37) on March 2, 2020 by immersing entire pots for 7 days in a tank with 2 cm water above the soil surface. After immersion, the pots were drained. The required amounts of K fertilizer were applied at the time of sowing. We applied 0.9 g and 0.375 g P pot^–1^ across all treatments, with 60% of N and 100% of P applied at the time of sowing. We applied 20% of N at the stem elongation stage (Zadoks Demical code 30) and 20% of N at the booting stage (Zadoks Demical code 43). The experiment used a randomized complete block design with a factorial arrangement. The two water treatments and two K levels were treated as the first and second independent factors, respectively. Ten replicate pots were allotted to each treatment, and pots with different treatments were rotated on a daily basis to ensure that all treatments had similar exposure to environmental factors.

Gas-exchange and chlorophyll fluorescence parameters were measured, and leaves, stems, and roots were sampled separately after the waterlogging treatment. Plants in four pots were harvested to provide four replicates. We mixed seven plants in each replicate pot and oven-dried them for 20 min at 105°C, and then at 75°C until constant weight. We averaged the dry weights of the seven plants to obtain a value for each replicate. At the beginning of anthesis (Zadoks Demical code 61), we separately marked the spikes blooming on each day. The anthesis date was obtained separately for each treatment when 50% of the spikes in the treatment had bloomed. After grain maturity, we harvested four pots to provide four replicates for each treatment. The number of spikes in each pot was recorded, and the spike number was calculated by dividing the number of spikes by the aperture area of the pot (0.05 m^2^). We threshed all of the spikes in each pot together and then recorded the grain number and the grain weight. The grain number per spike was calculated by dividing the grain number by the spike number. The 1,000-grain weight was calculated by dividing the grain weight by the grain number and then multiplying by 1,000. We calculated the grain yield by multiplying the spike number, the grain number per spike, and the 1,000-grian weight.

Supplementary Table S1 presents a schematic showing the developmental stages at which the K application and waterlogging treatments were applied, when the material was collected, and when the measurements were taken.

### Gas-Exchange, Chlorophyll Fluorescence, and Leaf Greenness Measurements

Pn, the intercellular CO_2_ concentration (*C*_i_), and stomatal conductance (*G*_s_) were measured during the morning from 09:00 to 11:00 using a gas exchange system (Li-Cor 6400; Li-Cor Inc., Lincoln, NE, United States). Leaf temperature, steady photosynthetic photon flux density (PPFD), reference CO_2_ in the cuvette, vapor pressure deficit (Vpdl), and relative humidity were maintained at 25.0 ± 0.5°C, 1,500 mol photons m^–2^ s^–1^, 400 ± 2.5 mol mol^–1^, 1.1 ± 0.05 kPa, and 55–65%, respectively.

Chlorophyll fluorescence was measured with a portable pulse amplitude modulation fluorescence monitoring system (PAM 2500; Walz, Effeltrich, Germany). The steady-state fluorescence (*F*_s_) was determined under actinic light. A saturating light pulse (∼8,000 mol photons m^–2^s^–1^) was applied to obtain maximum fluorescence (*F_m_’*) with light adaption under actinic light. After removing the actinic light and applying far-red light for 3 s, we obtained the minimal fluorescence of the light-adapted state (*F_o_*’). The minimum and maximum chlorophyll fluorescence (*F*_o_ and *F*_m_, respectively) were determined after full dark adaptation for at least 30 min. The quantum efficiency of photosystem II (PSII) (Φ*_PSII_*) and the maximum quantum efficiency of PSII (*F_v_/F_m_*) were calculated as $\frac{F_{m}^{\prime }-F_{s}}{F_{m}}$’ and $\frac{\left(F_{m}-F_{o}\right)}{F_{m}}$, respectively (Gao et al., 2018a).

Leaf greenness (SPAD readings) was measured with a chlorophyll meter (SPAD-502; Konica Minolta Sensing Inc., Osaka, Japan). We took SPAD readings from the center positions of the leaf blades.

We determined the gas-exchange, chlorophyll fluorescence, and leaf greenness values of the uppermost fully developed leaves after the waterlogging treatment. Measurements for each treatment were taken at 7–8 day intervals on the flag leaves of four plants in four replicate pots post-anthesis. To guarantee continuity during the leaf senescence process post-anthesis, we measured the gas-exchange, chlorophyll fluorescence, and leaf greenness of the flag leaves on the spikes blooming on the anthesis date.

### Element Analysis

Dried samples were ground to a fine powder, and four replicates were done for each treatment. The total N content was measured with an Elementar Vario EL Cube device (Elementar GmbH, Hanau, Germany). We acid-digested 100 mg of dried ground samples and two reference samples in 4 mL of concentrated HNO_3_: HClO_4_ (3:1). We then added deionized (DI) water to make up a total volume of 10 mL. Total P and K concentrations were determined with the 5300DV inductively coupled plasma-optical emission spectrometry (ICP-OES) device (Perkin Elmer, Shelton, CT, United States). Reference samples were included after each batch of 20 samples had been analyzed.

### Assessment of Leaf Senescence

We assessed leaf senescence based on the total duration of chlorophyll persistence and loss (Chl_total_), the duration of chlorophyll persistence (Chl_per_), and the duration of rapid chlorophyll loss (Chl_loss_), which were defined as the accumulated thermal time from anthesis to 90% senescence, from anthesis to 10% senescence, and from 10 to 90% senescence, respectively. We fitted the data for flag leaf chlorophyll over the accumulated thermal time post-anthesis using the Gompertz growth curve following Xie et al. (2016).

$$G=a\,e^{-b\,e^{\left(+r\,t\right)}}$$

where G is the average SPAD reading of four replicates, and t is the accumulated thermal time post-anthesis. We then calculated Chl_total_, Chl_per_, and Chl_loss_ as $\frac{\left(I\,n\,\left(\frac{I\,n\,\left(0.1\right)}{b}\right)\right)}{r}$, $\frac{\left(I\,n\,\left(\frac{I\,n\,\left(0.9\right)}{-b}\right)\right)}{r}$, and *Chl*_*total*_–*Chl*_*per*_, respectively.

### Statistical Analysis

The significance of the effects of waterlogging and K application was determined by two-way analysis of variance (ANOVA) using the SPSS 19.0 software (SPSS Inc., Chicago, IL, United States). Graphs were plotted using the SigmaPlot 10.0 software (Systat Software Inc., Chicago, IL, United States).

## Results

### Grain Yield and Yield Components

The grain yield in treatment W was significantly lower than the value in treatment C. However, grain yields were not significantly different among treatments C, C + K, and W + K (Table 1), indicating that yield reduction resulting from waterlogging was offset by increased K application. Treatment W decreased the 1,000-grain weight, but it did not affect the spike number or the grain number per spike.

**TABLE 1:** Effects of (i) waterlogging at the wheat stem elongation stage and (ii) potassium (K) application on spike number, grain number, 1,000-grain weight, and grain yield.

Treatment	Spike number (m^2^)	Grain number per spike	1,000-grain weight (g)	Grain yield (g m^2^)
C	491.7 ± 8.3^a^	44.2 ± 1.4^a^	45.5 ± 0.6^a^	989.5 ± 34.4^a^
W	458.3 ± 22.0^a^	46 ± 0.3^a^	34.4 ± 1.2^b^	723.2 ± 12.3^b^
C + K	483.3 ± 36.3^a^	46.3 ± 3.0^a^	44.6 ± 1.3^a^	992.4 ± 80.2^a^
W + K	475.0 ± 25.0^a^	43.8 ± 1.3^a^	46.6 ± 0.2^a^	968.3 ± 29.7^a^

*C, well-watered with 0.375 g K pot^–^^1^ (control); C + K, well-watered with 0.75 g K pot^–1^; W, waterlogging with 0.375 g K pot^–1^; W + K, waterlogging with 0.75 g K pot^–1^. Values are the means of four replicates. Lowercase letters indicate significant pairwise differences (P < 0.05 level, Dunnett’s multiple comparison test).*

### Morphological and Physiological Parameters After 7 Days of Waterlogging

**FIGURE 1:**
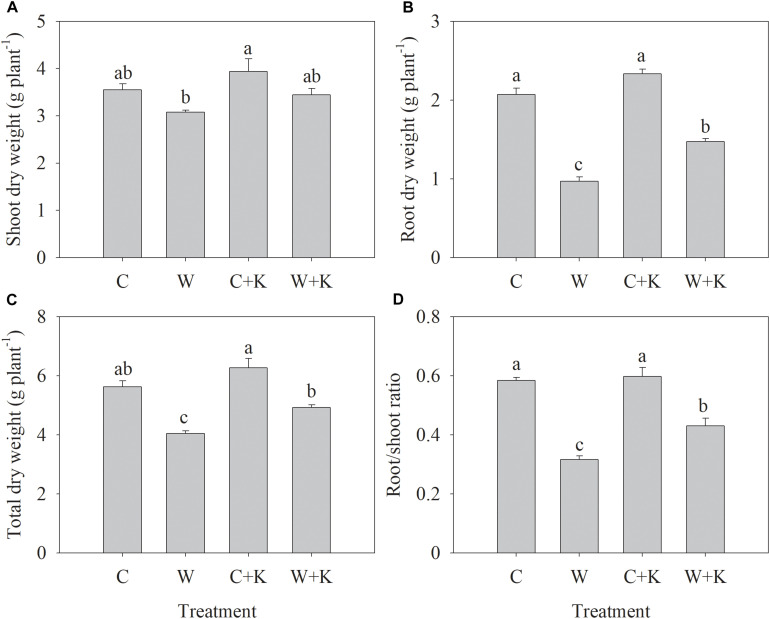
Effects of potassium (K) application on wheat shoot **(A)**, root **(B)**, and total **(C)** dry weights, and the root/shoot ratio **(D)** after waterlogging treatment. C, well-watered with 0.375 g K pot^–1^ (control); C + K, well-watered with 0.75 g K pot^–1^; W, waterlogging with 0.375 g K pot^–1^; W + K, waterlogging with 0.75 g K pot^–1^. Values are the means of four replicates. Lowercase letters indicate significant pairwise differences (*P* < 0.05 level, Dunnett’s multiple comparison test).

Waterlogging treatments (W and W + K) significantly decreased the root dry weight, the plant dry weight, and the root/shoot ratio to values below those in treatment C but did not have a significant effect on the shoot dry weight (Figure 1). However, the values of these parameters were higher in W + K plants than W plants, indicating that waterlogging-induced growth inhibition was mitigated by increasing K application.

**FIGURE 2:**
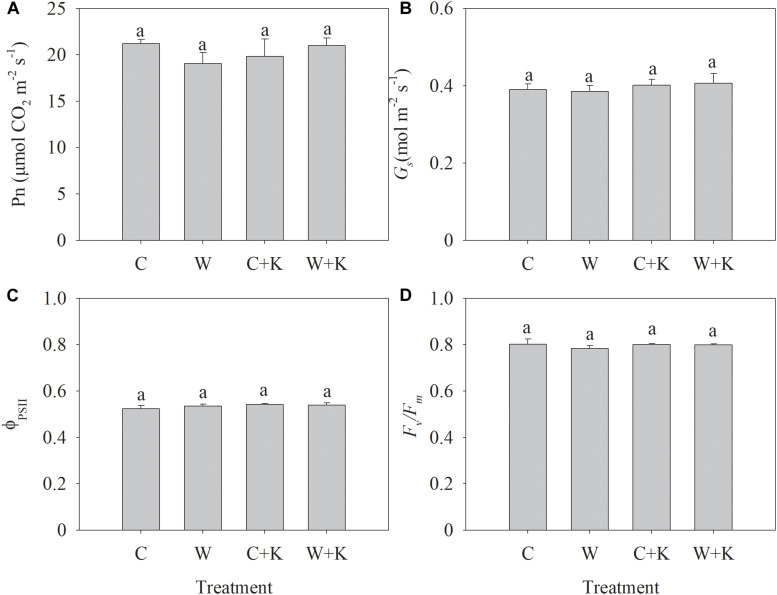
Effects of potassium (K) application on the net photosynthetic rate (Pn) **(A)**, stomatal conductance (*G*_s_) **(B)**, photochemical efficiency of photosystem II (PSII) (Φ*_PSII_*) **(C)**, and maximum quantum efficiency of PSII (*F_v_/F_m_*) **(D)** of wheat after waterlogging treatment. C, well-watered with 0.375 g K pot^–1^ (control); C + K, well-watered with 0.75 g K pot^–1^; W, waterlogging with 0.375 g K pot^–1^; W + K, waterlogging with 0.75 g K pot^–1^. Values are the means of four replicates. Lowercase letters indicate significant pairwise differences (*P* < 0.05 level, Dunnett’s multiple comparison test).

We found no significant difference in Pn, *G*_s_, Φ*_PSII_*, or *F_v_/F_m_* among treatments (Figure 2), indicating that waterlogging has negligible effects on photosynthesis.

**FIGURE 3:**
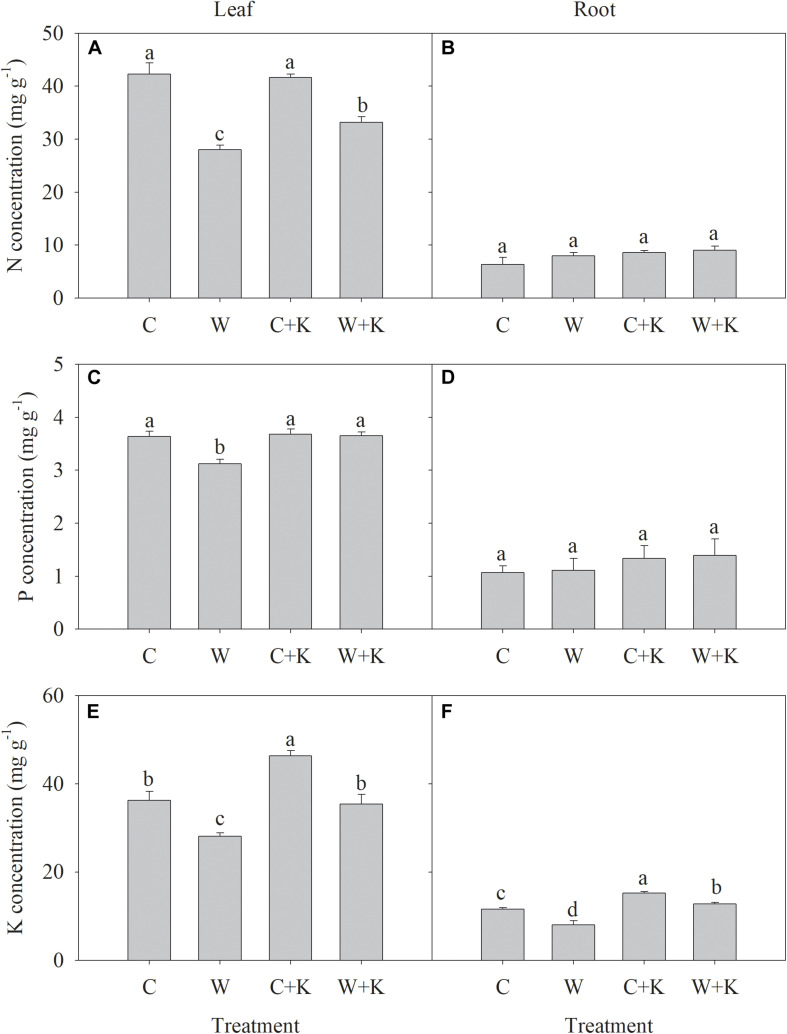
Effects of potassium (K) application on nitrogen (N) **(A,B)**, phosphorus (P) **(C,D)**, and K **(E,F)** concentrations in leaves and roots of wheat after waterlogging treatment. C, well-watered with 0.375 g K pot^–1^ (control); C + K, well-watered with 0.75 g K pot^–1^; W, waterlogging with 0.375 g K pot^–1^; W + K, waterlogging with 0.75 g K pot^–1^. Values are the means of four replicates. Lowercase letters indicate significant pairwise differences (*P* < 0.05 level, Dunnett’s multiple comparison test).

The N and P concentrations in leaves and roots were not significantly different between treatments C and C + K, but treatment C + K increased the K concentration in leaves and roots (Figure 3). Treatment W significantly reduced the N, P, and K concentrations in leaves and the K concentration in roots below values in treatment C but did not reduce the N and P concentrations in roots. However, the N, P, and K concentrations in leaves were significantly higher in W + K plants than in W plants.

### Photosynthesis Post-anthesis

The anthesis date was 1 day later in treatment W than in the other treatments (Table 2). Moreover, waterlogging reduced Chl_total_, Chl_per_, and Chl_loss_, indicating accelerated leaf senescence. However, the values of Chl_total_, Chl_per_, and Chl_loss_ were higher in treatment W + K than W.

**TABLE 2:** Effects of (i) waterlogging at the wheat stem elongation stage and (ii) potassium (K) application on the date of anthesis, total duration of chlorophyll persistence and loss (Chl_total_), duration of chlorophyll persistence (Chl_per_), and duration of rapid chlorophyll loss (Chl_loss_).

Treatment	Anthesis date	Chl_total_	Chl_per_	Chl_loss_
C	24.03.2020	568.3	395.4	172.9
W	25.03.2020	507.4	347.8	159.5
C + K	24.03.2020	574.7	417.1	157.6
W + K	24.03.2020	560.9	373.2	187.7

*C, well-watered with 0.375 g K pot^–1^ (control); C + K, well-watered with 0.75 g K pot^–1^; W, waterlogging with 0.375 g K pot^–1^; W + K, waterlogging with 0.75 g K pot^–1^. Values are the means of four replicates.*

**FIGURE 4:**
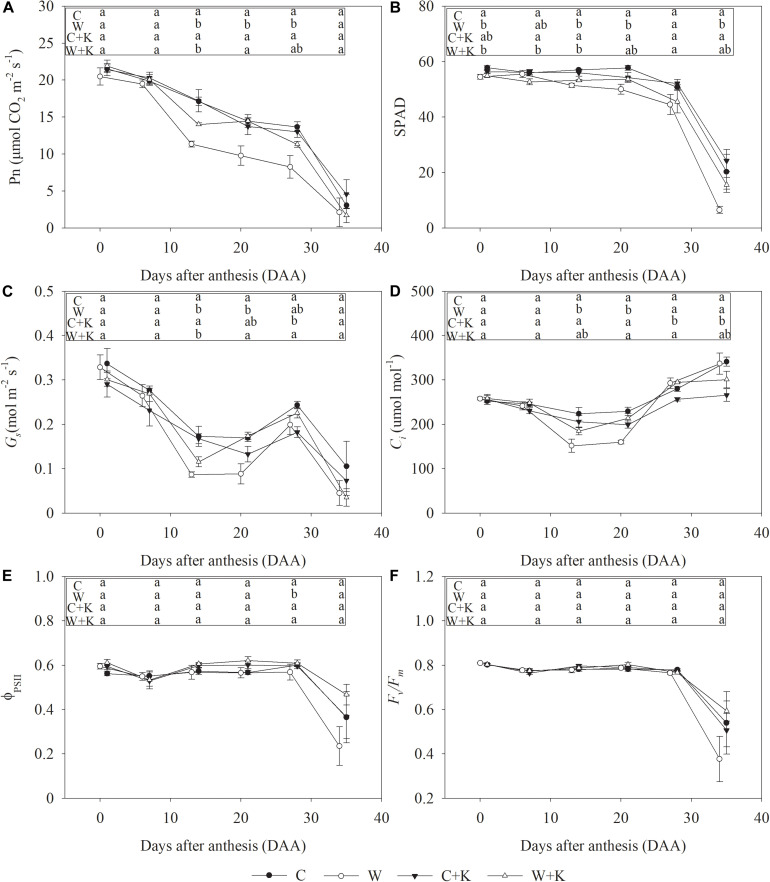
Effects of waterlogging and potassium (K) application on the net photosynthetic rate (Pn) **(A)**, leaf greenness (SPAD readings) **(B)**, stomatal conductance (*G*_s_) **(C)**, intercellular CO_2_ concentration (*C*_i_) **(D)**, photochemical efficiency of photosystem II (PSII) (Φ*_PSII_*) **(E)**, and maximum quantum efficiency of PSII (*F_v_/F_m_*) **(F)** of wheat after anthesis. C, well-watered with 0.375 g K pot^–1^ (control); C + K, well-watered with 0.75 g K pot^–1^; W, waterlogging with 0.375 g K pot^–1^; W + K, waterlogging with 0.75 g K pot^–1^. Values are the means of four replicates. Lowercase letters indicate significant pairwise differences (*P* < 0.05 level, Dunnett’s multiple comparison test).

A progressive decrease in Pn occurred post-anthesis. During days 7–21 after anthesis (DAA, days after anthesis), values of SPAD, Φ*_PSII_*, and *F_v_/F_m_* were not different from those on day zero, but *G*_s_ decreased dramatically, leading to a decrease in *C*_i_; this may account for the reduction in Pn during the early stage of leaf senescence post-anthesis. After 28 DAA, the SPAD, Φ*_PSII_*, and *F_v_/F_m_* values also decreased, but *C*_i_ increased, suggesting that non-stomatal factors accounted for the reduction in Pn during the late stage of leaf senescence post-anthesis (Figure 4).

Treatment W resulted in a significant reduction in Pn during the period 14–28 DAA. However, Pn was higher in treatment W + K than in treatment W, demonstrating that photosynthetic reduction due to waterlogging stress was mitigated by increased K application. On DAA 14 and 21, *G*_s_ and *C*_i_ values were reduced by the waterlogging treatment, but the reduction was mitigated by increased K application. Waterlogging led to a significant decrease in SPAD during the period 0–35 DAA, but it had no significant effects on Φ*_PSII_* or *F_v_/F_m_* during the period 0–21 DAA, suggesting that the reduction in leaf greenness caused by waterlogging did not influence photochemical efficiency during the early stage of leaf senescence. However, on DAA 28, the values of Φ*_PSII_* and *F_v_/F_m_* were reduced, while those of *C*_i_ were elevated in treatment W in comparison with treatment C, indicating that waterlogging caused a reduction in photochemical efficiency during the late stage of leaf senescence; however, this reduction was mitigated by increased K application.

## Discussion

Waterlogging among the major abiotic stressors constrains wheat yield (Setter and Waters, 2003; Hossain and Uddin, 2011; Manik et al., 2019). We found that waterlogging during the stem elongation reduced wheat yield by 27% (Table 1). The stem elongation stage is a key period in wheat grain yield formation because wheat spikes differentiate during this stage. The grain number is strongly affected by abiotic stresses in the period between spike initiation and anthesis, and the grain mass is reduced by stresses post-anthesis, e.g., heat stress (Gibson and Paulsen, 1994; Castro et al., 2007; Farooq et al., 2011). However, we found that 7 days of waterlogging at the stem elongation stage had no significant effect on the spike number or the grain number per spike but reduced the 1,000-grain weight (Table 1). Waterlogging also had negligible effects on shoot growth (Figure 1A) and photosynthesis (Figure 2) during the stem elongation stage. This may explain why waterlogging at the spike initiation stage did not reduce the grain number. However, waterlogging inhibited root growth significantly (Figure 1B), which may have affected plant growth and development in late developmental stages. Early anthesis and delayed leaf senescence promote increased individual grain weight (Xie et al., 2016). However, treatment W delayed the anthesis date by 1 day and accelerated leaf senescence, as indicated by the reduced values of Chl_total_, Chl_per_, and Chl_loss_ (Table 2). Araki et al. (2012) also concluded that wheat exposure to waterlogging at the jointing stage reduces Pn post-anthesis, thereby constraining grain filling. We found that waterlogging accelerated the decline in Pn post-anthesis (Figure 4A). Thus, waterlogging at the stem elongation stage led to early leaf senescence and photosynthetic degradation post-anthesis, thereby constraining grain filling; a reduction in the 1,000-grain weight was the sole cause of reduced yield.

The duration of photosynthesis in the flag leaf has major effects on grain yield (Carmo-Silva et al., 2017). Hence, attention should be focused on the mechanisms underlying the acceleration in photosynthetic rate reduction post-anthesis when waterlogging stress is imposed. Photosynthetic reduction is considered to be a result of leaf senescence: chlorophyll and photosynthetic proteins are recycled, and the chloroplasts break down during this period (Wingler et al., 1998). However, the converse also seems plausible because photosynthesis affects the production of carbohydrates and reactive oxygen species (ROS), which may serve as cues for leaf senescence initiation (Wang et al., 2015). We found that the decrease in Pn preceded the decreases in SPAD, Φ*_PSII_*, and *F_v_/F_m_* (Figures 4A,B,E,F). Therefore, Pn decreased before the breakdown of the photosynthetic apparatus began. Moreover, when the Pn decline began, *G*_s_ decreased significantly, which led to a decrease in *C*_i_ (Figures 4A,C,D). Stomatal movements are regulated by a sophisticated signal integration mechanism that includes multiple solutes, such as K^+^ and Ca^2+^, hormones, and ROS (Murata et al., 2015; Sierla et al., 2016). Cytokinins (CTK) play an important role in stomatal opening (Farber et al., 2016). They accumulate in grains at the early stage of development and influence grain size (Yang et al., 2000). During the early stages of grain development, grain and leaf competition for the CTK derived from roots may reduce foliar CTK, triggering stomatal closure. We found that waterlogging inhibited root growth (Figure 1), which may have, in turn, reduced foliar CTK and promoted the reductions in *G*_s_. CTK change in roots, leaves, and grains post-anthesis and their relations with *G*_s_ should be investigated in the future. *G*_s_ was consistently reduced by waterlogging to values below those in the control. Stomatal conductance is also related to leaf senescence signals, including ABA and ROS (Satler, 1979; Thimann and Satler, 1979; Wang et al., 2019). Therefore, the reduction in *G*_s_ under waterlogging treatment may have triggered early photosynthetic degradation post-anthesis.

*G*_s_ did not decrease further after DAA 14 (Figure 4C), whereas Pn continued to decline (Figure 4A), while *Ci* increased (Figure 4D), suggesting that non-stomatal factors accounted for the reduction in Pn (Li et al., 2015). No significant decline in SPAD, Φ*_PSII_*, or *F_v_/F_m_* occurred until DAA 35, demonstrating that photosynthetic apparatus disintegration occurred in the late senescence process (Figures 4B,E,F). Pn declined in the period 14–28 DAA, but Φ*_PSII_* did not; hence, there was a reduction in the CO_2_ assimilation pathway dissipation of energy captured by PSII. Treatment W significantly reduced Pn values in the period 14–28 DAA to lower values in the control. This can be explained by the low CO_2_ carboxylation capacity in treatment W that resulted from the low foliar N status (Figure 3A). Under these circumstances, excess excitation energy will trigger the production of ROS (Pascal et al., 2005). Alternatively, the excess excitation energy can be consumed by electron flux to the photorespiratory and Mehler pathways, which have important roles in photoprotection (Miyake and Yokota, 2000; Gao et al., 2018b, 2019). Φ*_PSII_* values in treatment W on DAA 28 were below those of the control. This effect may have been caused by the early shutdown of PSII (Kramer et al., 2004). Thus, waterlogging reduced PSII energy excitation flux to CO_2_ assimilation during late leaf senescence, increasing the risk of ROS generation, which may, in turn, disrupt the leaf senescence process. Under these circumstances, efforts to improve leaf N status and photoprotection capabilities could mitigate the reductions in photosynthesis caused by waterlogging.

We showed that increased K application attenuated the waterlogging-related effects of (i) a delayed anthesis date, (ii) early leaf senescence (Table 2), and (iii) acceleration of the decline in photosynthesis post-anthesis (Figure 4). Treatment K contributed to a higher 1,000-grain weight and reduced yield losses (Table 1). K has an important role in regulating stomatal function because the importation of K^+^ ions into guard cells required for stomatal opening (Fischer, 1968; Outlaw and Lowry, 1977). We showed that waterlogging reduced foliar [K] (Table 2), but increasing K application offsets this effect (Figure 3E). Furthermore, increased K application enhanced root growth significantly under waterlogging (Figure 1B and Table 2), which may, in turn, have promoted the root water absorption and CTK production that improved stomatal opening (Farber et al., 2016). Thus, in the period 14–21 DAA, *G*_s_ and *Ci* values were significantly higher in W + K-treated plants than in W-treated plants (Figure 4C). K may also affect the CO_2_ carboxylation capacity. Waterlogging significantly reduced foliar [N] and [P], which are crucial elements for the synthesis of photosynthetic enzymes, but increased K application mitigated these reductions (Figures 3A,C). K^+^ also facilitates well-structured stacking of stroma lamellae and grana, which enhances the efficiency of CO_2_ assimilation (Tränkner et al., 2018). Hence, increased K application may improve CO_2_ assimilation, thereby dissipating excess excitation energy and reducing the risk of photodamage. This mechanism may explain why the Pn values during the late senescence stage in W + K-treated plants were higher than those in W-treated plants (Figure 4A).

In conclusion, waterlogging at the stem elongation stage accelerated the decline in photosynthesis post-anthesis, which resulted in a reduction in the 1,000-grain weight and yield. The decrease in Pn during the early senescence stage due to waterlogging was attributable to stomatal closure; decreases during late senescence under waterlogging were attributable to a reduced CO_2_ carboxylation capacity, which increased the risk of photodamage. However, K had an important role in maintaining stomatal openings and CO_2_ carboxylation, thereby mitigating waterlogging-accelerated photosynthetic reductions and yield losses.

## Data Availability Statement

The raw data supporting the conclusions of this article will be made available by the authors, without undue reservation.

## Author Contributions

JG and AS planned and designed the research. JG, YS, MY, and YH performed experiments, conducted fieldwork, and analyzed data etc. JG, YS, MY, YH, and AS wrote the manuscript. All authors contributed to the article and approved the submitted version.

## Conflict of Interest

The authors declare that the research was conducted in the absence of any commercial or financial relationships that could be construed as a potential conflict of interest.

## Supplementary Material

Supplementary Table 1 List of dates and the developmental stages when (i) seeds were sown, (ii) K was applied, (iii) waterlogging treatments were applied, (iv) material was collected, (v) measurements were taken, and (vi) grain was harvested. Numbers in brackets are Zadoks decimal codes, which refer to the growth stages of cereals.

## Abbreviations

C: control treatment, well-watering + controversial K rate (0.375 g K pot^–1^)
C + K: well-watering + high K rate (0.75 g K pot^–1^)
Chl_total_: total duration of chlorophyll persistence and loss
Chl_per_: duration of chlorophyll persistence
Chl_loss_: duration of rapid chlorophyll loss
*C*_i_: intercellular CO_2_ concentration
*F*_m_: maximum chlorophyll fluorescence
*F_m_’*: maximum fluorescence of light-adapted state
*F*_o_: minimum chlorophyll fluorescence
*F_o_’*: minimal fluorescence of light-adapted state
*F*_s_: steady-state fluorescence
*F_v_/F_m_*: maximum quantum efficiency of photosystem II
*G*_s_: stomatal conductance
PCA: principal component analysis
Pn: net photosynthetic rate
SPAD: leaf greenness
Vpdl: vapor pressure deficit
W: waterlogging + controversial K rate
W + K: waterlogging + high K rate
Φ_PSII_: quantum efficiency of PSII.

## References

[B1] Araki H. Hamada A. Hossain M. A. Takahashi T. (2012). Waterlogging at jointing and/or after anthesis in wheat induces early leaf senescence and impairs grain filling. *Field Crops Res.* 137 27–36. 10.1016/j.fcr.2012.09.006

[B2] Camp P. J. Huber S. C. Burke J. J. Moreland D. E. (1982). Biochemical changes that occur during senescence of wheat leaves : I. Basis for the reduction of photosynthesis. *Plant Physiol.* 70 1641–1646. 10.1104/pp.70.6.1641 16662736 PMC1065947

[B3] Carmo-Silva E. Andralojc P. J. Scales J. C. Driever S. M. Mead A. Lawson T. (2017). Phenotyping of field-grown wheat in the UK highlights contribution of light response of photosynthesis and flag leaf longevity to grain yield. *J. Exp. Bot.* 68 3473–3486. 10.1093/jxb/erx169 28859373 PMC5853948

[B4] Castro M. Peterson C. J. Rizza M. D. Dellavalle P. D. Vázquez D. IbáÑez V. (2007). “Influence of heat stress on wheat grain characteristics and protein molecular weight distribution,” in *Wheat Production in Stressed Environments*, eds Buck H. T. Nisi J. E. Salomón N. (Dordrecht: Springer), 365–371. 10.1007/1-4020-5497-1_45

[B5] Celedonio R. P. D. S. Abeledo L. G. Miralles D. J. (2014). Identifying the critical period for waterlogging on yield and its components in wheat and barley. *Plant Soil* 378 265–277. 10.1007/s11104-014-2028-6

[B6] Chen Y. Huang J. Song X. Gao P. Wan S. Shi L. (2018). Spatiotemporal characteristics of winter wheat waterlogging in the middle and lower reaches of the yangtze river. China. *Adv. Meteorol.* 2018 1–11. 10.1155/2018/3542103

[B7] Cui J. Davanture M. Zivy M. Lamade E. Tcherkez G. (2019). Metabolic responses to potassium availability and waterlogging reshape respiration and carbon use efficiency in oil palm. *New Phytol.* 223 310–322. 10.1111/nph.15751 30767245

[B8] Diaz C. Lemaître T. Christ A. Azzopardi M. Kato Y. Sato F. (2008). Nitrogen recycling and remobilization are differentially controlled by leaf senescence and development stage in *Arabidopsis* under low nitrogen nutrition. *Plant Physiol.* 147 1437–1449. 10.1104/pp.108.119040 18467460 PMC2442554

[B9] Dreyer I. Gomez-Porras J. L. Riedelsberger J. (2017). The potassium battery: a mobile energy source for transport processes in plant vascular tissues. *New Phytol.* 216 1049–1053. 10.1111/nph.14667 28643868

[B10] Farber M. Attia Z. Weiss D. J. (2016). Cytokinin activity increases stomatal density and transpiration rate in tomato. *J. Exp. Bot.* 67 6351–6362. 10.1093/jxb/erw398 27811005 PMC5181579

[B11] Farooq M. Bramley H. Palta J. A. Siddique K. H. M. (2011). Heat stress in wheat during reproductive and grain-filling phases. *Crit. Rev. Plant Sci.* 30 491–507. 10.1080/07352689.2011.615687

[B12] Fischer R. A. (1968). Stomatal opening: role of potassium uptake by guard cells. *Science* 160 784–785. 10.1126/science.160.3829.784 5646418

[B13] Gao J. Luo Q. Sun C. Hu H. Wang F. Tian Z. (2019). Low nitrogen priming enhances photosynthesis adaptation to water-deficit stress in winter wheat (*Triticum aestivum* L) seedlings. *Front. Plant Sci.* 10:818. 10.3389/fpls.2019.00818 31293611 PMC6606716

[B14] Gao J. Wang F. Hu H. Jiang S. Muhammad A. Shao Y. (2018a). Improved leaf nitrogen reutilisation and Rubisco activation under short-term nitrogen-deficient conditions promotes photosynthesis in winter wheat (*Triticum aestivum* L.) at the seedling stage. *Funct. Plant Biol.* 45 840–853. 10.1071/fp17232 32291066

[B15] Gao J. Wang F. Sun J. Tian Z. Hu H. Jiang S. (2018b). Enhanced Rubisco activation associated with maintenance of electron transport alleviates inhibition of photosynthesis under low nitrogen conditions in winter wheat seedlings. *J. Exp. Bot.* 69 5477–5488. 30239847 10.1093/jxb/ery315

[B16] Gibson L. R. Paulsen G. M. (1994). Yield components of wheat grown under high temperature stress during reproductive growth. *Crop Sci.* 39 1841–1846. 10.2135/cropsci1999.3961841x

[B17] Herzog M. Striker G. G. Colmer T. D. Pedersen O. (2016). Mechanisms of waterlogging tolerance in wheat–a review of root and shoot physiology. *Plant Cell Environ.* 39 1068–1086. 10.1111/pce.12676 26565998

[B18] Hossain A. Uddin S. N. (2011). Mechanisms of waterlogging tolerance in wheat: Morphological and metabolic adaptations under hypoxia or anoxia. *Austr. J. Crop Sci.* 5 1094–1101.

[B19] Kramer D. M. Johnson G. Kiirats O. Edwards G. E. (2004). New fluorescence parameters for the determination of QA redox state and excitation energy fluxes. *Photosynth. Res.* 79 209–218. 10.1023/b:pres.0000015391.99477.0d 16228395

[B20] Li H. Wang Y. Xiao J. Xu K. (2015). Reduced photosynthetic dark reaction triggered by ABA application increases intercellular CO2 concentration, generates H2O2 and promotes closure of stomata in ginger leaves. *Environ. Exp. Bot.* 113 11–17. 10.1016/j.envexpbot.2015.01.002

[B21] Manik S. N. Pengilley G. Dean G. Field B. Shabala S. Zhou M. (2019). Soil and crop management practices to minimize the impact of waterlogging on crop productivity. *Front. Plant Sci.* 10:140. 10.3389/fpls.2019.00140 30809241 PMC6379354

[B22] Masclaux C. Valadier M.-H. Brugière N. Morot-Gaudry J.-F. Hirel B. (2000). Characterization of the sink/source transition in tobacco (*Nicotiana tabacum* L.) shoots in relation to nitrogen management and leaf senescence. *Planta* 211 510–518. 10.1007/s004250000310 11030550

[B23] Miyake C. Yokota A. (2000). Determination of the rate of photoreduction of O2 in the water-water cycle in watermelon leaves and enhancement of the rate by limitation of photosynthesis. *Plant Cell Physiol.* 41 335–343. 10.1093/pcp/41.3.335 10805597

[B24] Murata Y. Mori I. C. Munemasa S. (2015). Diverse stomatal signaling and the signal integration mechanism. *Annu. Rev. Plant Biol.* 66 369–392. 10.1146/annurev-arplant-043014-114707 25665132

[B25] Nguyen L. T. Osanai Y. Anderson I. C. Bange M. P. Braunack M. Tissue D. T. (2018). Impacts of waterlogging on soil nitrification and ammonia-oxidizing communities in farming system. *Plant Soil* 426 299–311. 10.1007/s11104-018-3584-y

[B26] Noreen S. Fatima Z. Ahmad S. Ashraf M. (2018). “Foliar application of micronutrients in mitigating abiotic stress in crop plants,” in *Plant Nutrients and Abiotic Stress Tolerance*, eds Hasanuzzaman M. Fujita M. Oku H. Nahar K. Hawrylak-Nowak B. (Singapore: Springer), 95–117. 10.1007/978-981-10-9044-8_3

[B27] Outlaw W. H. Lowry O. H. (1977). Organic acid and potassium accumulation in guard cells during stomatal opening. *Proc. Nat. Acad. Sci. U.S.A.* 74 4434–4438. 10.1073/pnas.74.10.4434 16592449 PMC431957

[B28] Pascal A. A. Liu Z. Broess K. van Oort B. van Amerongen H. Wang C. (2005). Molecular basis of photoprotection and control of photosynthetic light-harvesting. *Nature* 436 134–137. 10.1038/nature03795 16001075

[B29] Romina P. Abeledo L. G. Miralles D. J. (2018). Physiological traits associated with reductions in grain number in wheat and barley under waterlogging. *Plant Soil* 429 469–481. 10.1007/s11104-018-3708-4

[B30] Satler T. S. (1979). Relation between senescence and stomatal opening: senescence in darkness. *Proc. Natl. Acad. Sci. U.S.A.* 76 2770–2773. 10.1073/pnas.76.6.2770 16592665 PMC383690

[B31] Setter T. Waters I. (2003). Review of prospects for germplasm improvement for waterlogging tolerance in wheat, barley and oats. *Plant Soil* 253 1–34. 10.1023/a:1024573305997

[B32] Sierla M. Waszczak C. Vahisalu T. Kangasjarvi J. (2016). Reactive oxygen species in the regulation of stomatal movements. *Plant Physiol.* 171 1569–1580. 10.1104/pp.16.00328 27208297 PMC4936562

[B33] Singh S. P. Setter T. L. (2017). Effect of waterlogging on element concentrations, growth and yield of wheat varieties under farmer’s sodic field conditions. *Proc. Natl. Acad. Sci. Sect. B Biol. Sci.* 87 513–520. 10.1007/s40011-015-0607-9

[B34] Thimann K. V. Satler S. O. (1979). Relation between leaf senescence and stomatal closure: Senescence in light. *Proc. Natl. Acad. Sci. U.S.A*, 76 2295–2298. 10.1073/pnas.76.5.2295 16592651 PMC383586

[B35] Tränkner M. Tavakol E. Jákli B. (2018). Functioning of potassium and magnesium in photosynthesis, photosynthate translocation and photoprotection. *Physiol. Plant.* 163 414–431. 10.1111/ppl.12747 29667201

[B36] Trnka M. Rötter R. P. Ruiz-Ramos M. Kersebaum K. C. Olesen J. E. Žalud Z. (2014). Adverse weather conditions for European wheat production will become more frequent with climate change. *Nat. Clim. Change* 4 637–643. 10.1038/nclimate2242

[B37] Wang H. Yan S. Xin H. Huang W. Zhang H. Teng S. (2019). A subsidiary cell-localized glucose transporter promotes stomatal conductance and photosynthesis. *Plant Cell* 31 1328–1343. 10.1105/tpc.18.00736 30996077 PMC6588317

[B38] Wang J. Leister D. Bolle C. (2015). Photosynthetic lesions can trigger accelerated senescence in Arabidopsis thaliana. *J. Exp. Bot.* 66 6891–6903. 10.1093/jxb/erv393 26272903 PMC4623695

[B39] Wingler A. Von Schaewen A. Leegood R. C. Lea P. J. Quick W. P. (1998). Regulation of leaf senescence by cytokinin, sugars, and light effects on NADH-dependent hydroxypyruvate reductase. *Plant Physiol.* 116 329–335. 10.1104/pp.116.1.329

[B40] Wollmer A. C. Pitann B. Mühling K. H. (2018). Nutrient deficiencies do not contribute to yield loss after waterlogging events in winter wheat (*Triticum aestivum*). *Ann. Appl. Biol.* 173 141–153. 10.1111/aab.12449

[B41] Xie Q. Mayes S. Sparkes D. L. (2016). Early anthesis and delayed but fast leaf senescence contribute to individual grain dry matter and water accumulation in wheat. *Field Crops Res.* 187 24–34. 10.1016/j.fcr.2015.12.009

[B42] Yang J. Peng S. Visperas R. M. Sanico A. L. Zhu Q. Gu S. (2000). Grain filling pattern and cytokinin content in the grains and roots of rice plants. *Plant Growth Regul.* 30 261–270.

