# Supplementary material for: Potassium Alleviates Post-anthesis Photosynthetic Reductions in Winter Wheat Caused by Waterlogging at the Stem Elongation Stage
Source: Front Plant Sci. 2021 Jan 12;11:607475. doi: 10.3389/fpls.2020.607475 (PMC7835391; doi:10.3389/fpls.2020.607475)
Supplement: Supplementary Table 1 — List of dates and the developmental stages when (i) seeds were sown, (ii) K was applied, (iii) waterlogging treatments were applied, (iv) material was collected, (v) measurements were taken, and (vi) grain was harvested. Numbers in brackets are Zadoks decimal codes, which refer to the growth stages of cereals. [file Table_1.DOCX]

Table.S1. List of dates and the developmental stages when (i) seeds were sown, (ii) K was applied, (iii) waterlogging treatments were applied, (iv) material was collected, (v) measurements were taken, and (vi) grain was harvested. Numbers in brackets are Zadoks decimal codes, which refer to the growth stages of cereals.

| Date | C | W | C+K | W+K |
| --- | --- | --- | --- | --- |
| 22-Nov-19 | **Sowing** 0.375 g K pot-1 as basal fertilizer | **Sowing**  0.375 g K pot-1 as basal fertilizer | **Sowing**  0.75 g K pot-1 as basal fertilizer | **Sowing**  0.75 g K pot-1 as basal fertilizer |
| 2-Mar-20 | **Stem elongation stage (37)**  Well watering treatment | **Stem elongation stage (37)**  Waterlogging treatment | **Stem elongation stage (37)**  Well watering treatment | **Stem elongation stage (37)**  Waterlogging treatment |
| 9-Mar-20 | **Stem elongation stage (39)**  Sampling, gas-exchange and chlorophyll fluorescence measurements | **Stem elongation stage (39)** Drainage, sampling, gas-exchange and chlorophyll fluorescence measurements | **Stem elongation stage (39)**  Sampling, gas-exchange and chlorophyll fluorescence measurements | **Stem elongation stage (39)** Drainage, sampling, gas-exchange and chlorophyll fluorescence measurement**s** |
| 24-Mar-20 | **Anthesis date(61)**  50% of the spikes in this treatment bloomed |  | **Anthesis date(61)** 50% of the spikes in this treatment bloomed | **Anthesis date(61)** 50% of the spikes in this treatment bloomed |
| 25-Mar-20 | **1 Days after anthesis**  Gas-exchange, chlorophyll fluorescence and SPAD measurements | **Anthesis date(61)** Gas-exchange, chlorophyll fluorescence and SPAD measurements | **1 Days after anthesis**  Gas-exchange, chlorophyll fluorescence and SPAD measurements | **1 Days after anthesis**  Gas-exchange, chlorophyll fluorescence and SPAD measurements |
| 31-Mar-20 | **7 Days after anthesis**  Gas-exchange, chlorophyll fluorescence and SPAD measurements | **6 Days after anthesis**  Gas-exchange, chlorophyll fluorescence and SPAD measurements | **7 Days after anthesis**  Gas-exchange, chlorophyll fluorescence and SPAD measurements | **7 Days after anthesis**  Gas-exchange, chlorophyll fluorescence and SPAD measurements |
| 7-Apr-20 | **14 Days after anthesis**  Gas-exchange, chlorophyll fluorescence and SPAD measurements | **13 Days after anthesis**  Gas-exchange, chlorophyll fluorescence and SPAD measurements | **14 Days after anthesis**  Gas-exchange, chlorophyll fluorescence and SPAD measurements | **14 Days after anthesis**  Gas-exchange, chlorophyll fluorescence and SPAD measurements |
| 14-Apr-20 | **21 Days after anthesis**  Gas-exchange, chlorophyll fluorescence and SPAD measurements | **20 Days after anthesis**  Gas-exchange, chlorophyll fluorescence and SPAD measurements | **21 Days after anthesis**  Gas-exchange, chlorophyll fluorescence and SPAD measurements | **21 Days after anthesis**  Gas-exchange, chlorophyll fluorescence and SPAD measurements |
| 21-Apr-20 | **28 Days after anthesis**  Gas-exchange, chlorophyll fluorescence and SPAD measurements | **27 Days after anthesis**  Gas-exchange, chlorophyll fluorescence and SPAD measurements | **28 Days after anthesis**  Gas-exchange, chlorophyll fluorescence and SPAD measurements | **28 Days after anthesis**  Gas-exchange, chlorophyll fluorescence and SPAD measurements |
| 28-Apr-20 | **35 Days after anthesis**  Gas-exchange, chlorophyll fluorescence and SPAD measurements | **34 Days after anthesis**  Gas-exchange, chlorophyll fluorescence and SPAD measurements | **35 Days after anthesis**  Gas-exchange, chlorophyll fluorescence and SPAD measurements | **35 Days after anthesis**  Gas-exchange, chlorophyll fluorescence and SPAD measurements |
| 4-May-20 | **Ripening (99)** Harvest | **Ripening (99)** Harvest | **Ripening (99)** Harvest | **Ripening (99)** Harvest |
